# BAG3 Suppresses Loading of Ago2 to IL6 mRNA in Pancreatic Ductal Adenocarcinoma

**DOI:** 10.3389/fonc.2019.00225

**Published:** 2019-04-02

**Authors:** Chao Li, Ming-Xin An, Jing-Yi Jiang, Han-Bing Yao, Si Li, Jing Yan, Xin-Yu Li, Hua-Qin Wang

**Affiliations:** ^1^Department of Biochemistry and Molecular Biology, China Medical University, Shenyang, China; ^2^Key Laboratory of Cell Biology, Ministry of Public Health, and Key Laboratory of Medical Cell Biology, Ministry of Education, China Medical University, Shenyang, China

**Keywords:** BAG3, IL6, Ago2, pancreatic cancer, fibrosis

## Abstract

Pancreatic stellate cells (PSCs) are a subset of pancreatic cancer-associated fibroblasts, which play a critical role in pancreatic fibrosis, a characteristic feature of pancreatic cancer. The interplay between PSCs and pancreatic cancer cells is vital for promotion of tumor progression and metastasis. BAG3 is correlated with poor prognostics in patients with pancreatic ductal adenocarcinoma (PDAC), however, the exact mechanisms remain largely unknown. In this study, we demonstrated that BAG3 downregulation decreased IL6 release by PDACs, and IL6 reduction was, at least partially, responsible for suppression of PSCs activation by PDACs with BAG3 downmodulation. Importantly, BAG3 expression positively correlated with fibrosis in pancreatic cancer tissue. With regard to the underlying mechanism, we demonstrated that BAG3 knockdown facilitated recruitment of Agonaute 2 (Ago2) to IL6 mRNA, resulting in destabilization of IL6 mRNA. In addition, the current study demonstrated that phosphorylation at Serine (Ser) 387 site was required for recruitment of Ago2-containing miRISC to IL6 mRNA and BAG3 knockdown facilitated Ago2 loading to IL6 mRNA via increasing its phosphorylation at Ser 387. This study shed new light on the tumor-promoting role of BAG3 in PDAC tumors, suggesting BAG3 might represent an interesting therapeutic opportunity to PDAC patients.

## Introduction

Pancreatic ductal adenocarcinoma (PDAC) reveals highly malignant phenotypes including rapid progression and early metastasis (1). The microenvironment of human PDAC is characterized by a dense fibrotic stroma (desmoplasia) generated by excessive stromal cells (2). The source of the fibrotic stroma has been extensively explored, and it has been established that pancreatic stellate cells (PSCs) are the primary stromal cells in the tumor microenvironment of PDAC tumors. PSCs remain in a quiescent state in normal pancreas, however, they can transform to an activated state under various pathological conditions, such as cancer and inflammatory disease (3, 4). Activated PSCs acquire a myofibroblast like phenotype, which can proliferate and migrate (5). Activated PSCs also synthesize various cytokines and extracellular matrix components, and play a pivotal role in fibrotic extracellular environment in pancreatic cancer (3, 4). In recent years, the critical role of the interaction between PSCs and pancreatic cancer cells in PDAC tumors has been increasingly recognized. Pancreatic cancer cells can drive PSCs into an activated state via direct interaction or via release of growth factors (6), and in turn, PSCs enhance the malignancy of pancreatic cancer via releasing a large number of cytokines and extracellular matrix proteins (7–11). Therefore, attenuation of PSC activation may be a promising therapeutic approach for pancreatic cancer in the future (12). However, the mechanisms underlying PSC activation are yet to be discovered.

BAG3 is a member of the family of heat shock protein (HSP) 70 co-chaperones that share the evolutionarily conserved BAG domain (13). BAG3 expression is limited to few cell types such as myocytes in physiological conditions (14), while its expression is constitutively expressed in a wide range of human tumors (15–23). BAG3 is assigned a role in sustaining the growth of some tumor types and its expression is correlated with the poor prognosis of some cancers including PDAC (24–26). However, the oncogenic potential of BAG3 remains incompletely understood.

Non-coding RNAs have been shown to regulate the expression of protein-coding genes either transcriptionally or post-transcriptionally (27, 28). To exert their function, the non-coding RNAs interact with one or more members of Argonaute (Ago) family, which are essential effectors of RNA-induced silencing complex (RISC). Although all four Ago proteins have been implicated in translational inhibition of mRNA, only Ago2 possesses endoribonuclease activity, thus functions as an essential effector in RNA-induced silencing complex (RISC) (27, 28).

Here we show that BAG3 expression positively correlates with fibrotic microenvironment in PDAC tissues. Importantly, BAG3 downregulation in PDAC cells suppressed their capacity to activate PSCs. Mechanistically, we demonstrated that BAG3 knockdown weakened the ability of PDACs to drive PSCs activation via destabilizing IL6 mRNA in an Ago2-dependent manner. Secreted by various cells like tumor and fibroblast cells, monocytes, and macrophages, IL6 has been reported to be one of the major factors responsible for PSCs activation (29, 30). This in turn will lead to a rational basis for combination strategies that will include BAG3 silencing/inhibition in PDAC therapy.

## Materials and Methods

### Knockdown of BAG3 by CRISPR/Cas9

A dual gRNA approach was used to knockdown BAG3 by CRISPR/Cas9 system. To facilitate the selection of positive clones, a donor vector was generated in such a way that targeting sequence is replaced by marker genes (GFP and PU, the puromycin resistance gene) once it is integrated into the genomic DNA by homologous recombination. The dual gRNA construct carrying Cas9 and donor vector were introduced into PDAC cells by infection. The empty dual gRNA vector served as a control. The efficiency of gRNA was initially identified by genomic PCR, followed by real-time RT-PCR and Western blot.

### Human Primary Pancreatic Stellate Cells (HPanSteC)

Human primary pancreatic stellate cells (HPanSteC) were purchased from ScienCell company and maintained in Stellate Cell Medium as provider suggested.

### Pancreatic Cancer Cell Culture and Conditional Medium (CM)

BxPC3 and SW1990 pancreatic cancer cells were maintained in Dulbecco's modified Eagle's medium (DMEM) supplemented with 10% fetal bovine serum (FBS) and antibiotics (100 U/ml penicillin and 100 mg/ml streptomycin). Conditional medium (CM) was obtained by culturing cells to 70–80% confluence, washing twice and changing to serum-free media (SFM) containing 0.5% bovine serum albumin (BSA). CM was collected after 72 h incubation and aliquots stored at −80°C until further use.

### ELISA

Cytokine production in cell culture supernatants was quantified using a human IL6 (eBioscience, BMS213HS) immunoassay kit. Briefly, cells were cultured in serum free medium (SFM) containing 0.5% BSA. CM was collected after 72 h and used for analysis of IL6 release. Viable cell count confirmed that 0.5% BSA containing DMEM had no effect on cell viability during 72 h culture. IL6 release was normalized by cell numbers.

### Antibody

Mouse or rabbit IgG was purchased from GeneTex. Rabbit anti human BAG3 antibody was purchased from GeneText, mouse anti human α-SMA was purchased from Merck Millipore Company.

### Growth Curve and Migration Assays Using Real-Time Cell Analyzer (RTCA)

Growth curve and migration assays were performed in real time in quadruplicate with the xCELLigence system (ACEA Bioscience, San Diego, CA) as previously described (31).

### Transwell Migration Assays

*In vitro* Transwell migration assays were performed in modified Boyden chambers with 8 mm pore filter inserts in 24-well plates (BD Biosciences, San Jose, CA, USA). Briefly, the lower chamber was filled with DMEM containing 10% fetal bovine serum. HPanSteC Cells were collected after trypsinization, resuspended in 200 ml of conditional medium collected from PDACs, and transferred to the upper chamber. After 24 h of incubation, the filter was gently removed from the chamber, and the cells on the upper surface were removed using a cotton swab. Cells were stained with crystal violet.

### IL6 Neutralization Assay

To block the effects of IL6, cultured PDAC supernatants or recombinant IL6 were pretreated with 5 μg/ml of anti-IL6 antibody (clone 6708, R&D Systems) for 30 min before addition to PSC.

### Determination of mRNA Half-Life

To measure the half-life of endogenous IL6 mRNA, actinomycin D or Amanitin was added into the cell culture medium and total RNA was prepared at the times indicated and subjected to RT-qPCR analysis. For IL6, the forward primer was 5′-TACATCCTCGACGGCATCTCAG-3′ and the revers was 5′-TGCACAGCTCTGGCTTGTTCC-3′, the amplicon size is 257 bp. For β-actin, the forward primer was 5′-GAGACCTTCAACACCCCAGCC-3′ and the revers was 5′-GGATCTTCATGAGGTAGTCAG-3′, the amplicon size is 205 bp. IL6 mRNA levels were normalized to β-actin and plotted as a percentage of the value at time zero (set at 100%).

### Edu Incorporation Assays

*De novo* DNA synthesis was measured by Click-iT Edu Assay Kit (Invitrogen, Carlsbad, CA) according to the manufacturer's instructions. Briefly, cells were exposed to 10 μM of nucleoside analog 5-thynyl-2' deoxyuridine (Edu) for 4 h. Incorporated Edu was labeled using Alexa Fluor 555 azide in the provided reaction buffer for 30 min, then the nuclei was counterstained with DAPI.

### RNA Immunoprecipitation (RIP)

Magna RIP™ RNA-binding protein immunoprecipitation kit (Millipore) was used for RIP procedures according to the manufacturer's protocol. BAG3, Ago2, AUF1, HuR, TTP, or KSRP antibody was used to pull down IL6 mRNA. After the antibody was recovered by protein A/G beads, standard qRT-PCR was performed to detect HK2 mRNA in the precipitates.

### Generation of Reporter Vectors and Dual-Luciferase Reporter Assay

The 5′UTR (untranslational region), CR (coding region), and 3′UTR fragments of IL6 mRNA was generated by PCR and inserted into the pMIR-REPORT™ Luciferase vector (Promega, Madison, WI) just after the stop codon. The transfection was carried out with Lipofectamine 2000 (Invitrogen) according to the manufacturer's instructions. Cells were incubated for 48 h and harvested by adding 100 μl of reporter lysis buffer (Dual-Luciferase Assay System, Promega). The firefly and *Renilla* luciferase activities were then measured using the Dual-Luciferase Reporter Assay System (Promega, Madison, WI) and a luminometer (Mannedorf, Switzerland). Firefly luciferase (FFL) activities were normalized by Renilla (RL) activities yielding relative activities (RLU). All experiments were done in triplicate and independently performed at least three times to confirm the results. The mean ± SD calculated from one representative experiment was presented.

### Biotin Pull-Down Assay

Luciferase vector containing 3′UTR of IL6 mRNA was used as a template for the PCR amplification. All 5′ primers contained the T7 promoter sequence CCAAGCTTCTAATACGACTCACTATAGGGAG-3′ (T7). For biotin pulldown assays, PCR-amplified DNA was used as the template to transcribe biotinylated RNA by using T7 RNA polymerase in the presence of biotin-UTP. RNA-protein binding reactions were performed using 500 μg of cell lysates and 1 μg biotin-labeled RNA in a final volume of 20 μl using Binding Buffer A(20 mM HEPES-KOH at pH7.5, 2.5 mM magnesium chloride (MgCl_2_), 100 mM KCl, 20% glycerol, 0.5 mM dithiotheritol and protease inhibitor tablets). Reaction mixtures were incubated for 1 h at room temperature. Complexes were isolated with paramagnetic streptavidin-conjugated Dynabeads (Invitrogen), and the pulldown materials were analyzed by western blotting analysis.

### Label and Capture Nascent RNA

Newly synthesized RNA was labeled and isolated using Click-iT Nascent RNA Capture Kit (Invitrogen) as previously reported (32). Briefly, nascent RNAs were labeled with 0.2 mM of 5-ethymyl uridine (EU), followed by biotinylation and isolation using streptavidin magnetic beads.

### Western Blot Analysis

Total cellular proteins were extracted using lysis buffer containing 20 mM Tris-HCl, 150 mM NaCl, 2 mM EDTA, 1% Triton-X100 and protease inhibitor cocktail (Sigma-Aldrich, Saint Louis, MO). Extracted proteins were quantified using the BCA protein assay kit. Thirty microgram of total proteins was separated using 12% SDS-PAGE and transferred to PVDF membrane (Millipore Corporation, Billerica, MA).

### Tissue Microarray and Immunohistochemical Staining

Tissue microarray sections were purchased from Shanghai Outdo Biotech Co., LTD. Tissue sections was immunostained with antibodies to BAG3 and αSMA. A semi-quantitative H-score ranged was calculated for each specimen by multiplying the distribution areas (0–100%) at each staining intensity level by the intensities (0: negative; 1: weak staining; 2: moderate staining; 3: strong staining) as previously reported (33).

### Statistics

The statistical significance of the difference was analyzed by ANOVA and *post-hoc* Dunnett's test. Statistical significance was defined as *P* < 0.05. All experiments were repeated three times, and data were expressed as the mean ± SD (standard deviation) from a representative experiment.

## Results

### BAG3 Knockdown Decreased IL6 Production in PDACs and Compromised Activation of HPanSteC by PDACs

**Figure 1:**
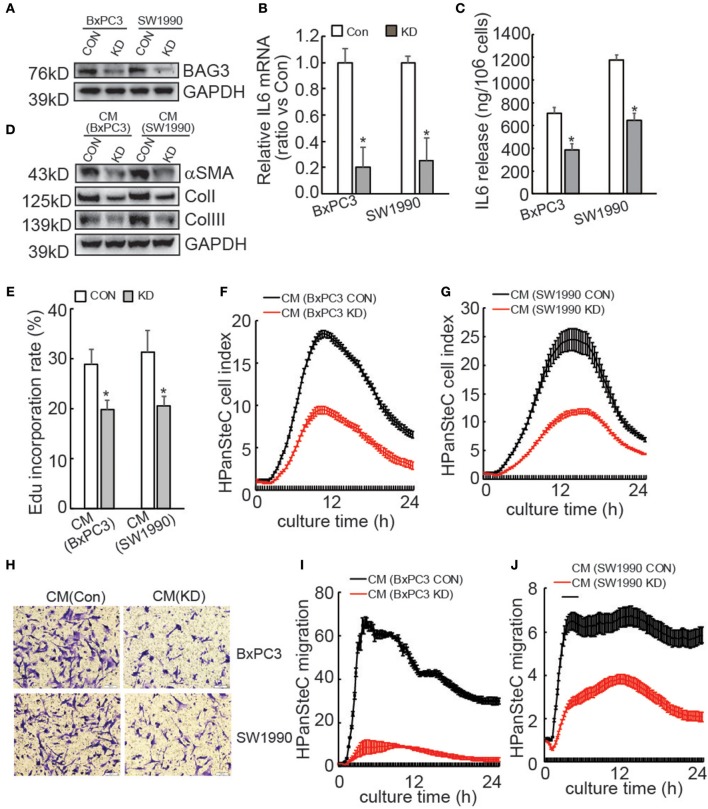
Activation of PSCs is compromised by supernatants of PDACs with BAG3 knockdown. **(A)** BxPC3 or SW1990 cells were infected with control (CON) or gRNA guided BAG3 (KD) using CRISPR/Cas9 system, BAG3 expression was confirmed using Western blot. **(B)** IL6 mRNA expression was analyzed in the indicated cells using real-time PCR. **(C)** IL6 extracellular release was analyzed in the indicated cells using ELISA and standardized the control and the experimental group by cell numbers. **(D–J)** HPanSteC cells were incubated with conditional medium (CM) collected from BxPC3 or SW1990, αSMA, and collagen expression was analyzed using Western blot analysis **(D)**, DNA synthesis was analyzed using Edu incorporation **(E)**, cell proliferation was analyzed using RTCA **(F,G)**, cell migration was analyzed using Transwell **(H)**, and RTCA **(I,J)**, respectively. *P < 0.01.

We and others have reported that BAG3 expression is an independent poor prognostic factor with respect to overall survival in patients with pancreatic cancer (25, 34). To explore the potential mechanisms underlying oncogenic function of BAG3 in PDACs, we screened differential mRNA expression by BAG3 knockdown in BxPC3 cells (Supplementary Data 1). Among differentially expressed gene, downregulation of IL6 by BAG3 knockdown attracted our attention, as IL6 is one of key player in pancreatic fibrosis. We confirmed that knockdown of BAG3 (Figure 1A) decreased IL6 mRNA levels (Figure 1B) and IL6 release to supernatant (Figure 1C) in both BxPC3 and SW1990 cells. We then investigated whether BAG3 expression in PDACs might affect activation of HPanSteC, human primary pancreatic stellate cells. Western blot demonstrated that compared with cells incubated with CM derived from control PDAC cells, expression of αSMA and Collagens was markedly decreased in HPanSteC cells incubated with CM derived from PDAC cells with BAG3 knockdown (Figure 1D). Edu incorporation (Figure 1E) and RTCA proliferation assay (Figures 1F,G) demonstrated that proliferation of HPanSteC cells was slowed down upon incubation with CM derived from PDACs with BAG3 knockdown, when compared with that from their control partners. Transwell (Figure 1H) and RTCA (Figures 1I,J) migration assays demonstrated that HPanSteC cells incubated with CM from PDACs with BAG3 knockdown migrated slower than those incubated with CM derived from control PDACs.

**Figure 2:**
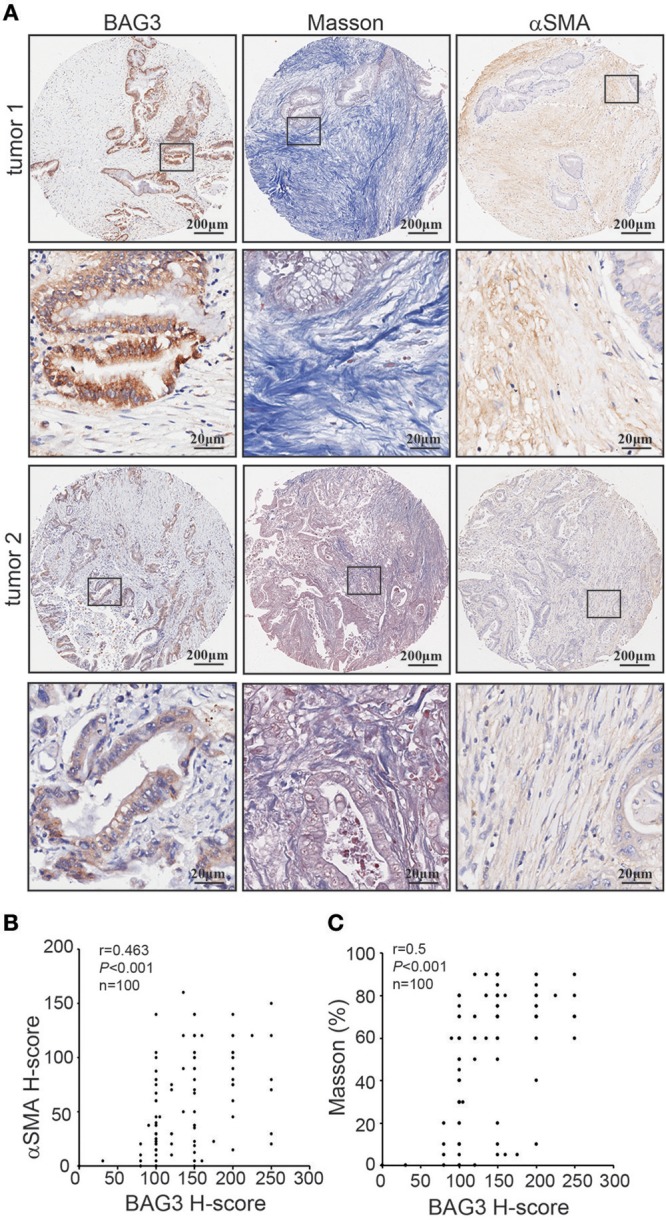
Correlation of BAG3 and fibrosis in pancreatic cancer tissues. **(A)** Representative immunohistochemistry staining with BAG3, αSMA, as well as Masson staining in pancreatic cancer tissues. **(B,C)** Scatter plots showing the positive correlation between BAG3 and αSMA **(B)** or Masson **(C)** IHC score in pancreatic cancer tissues. Pearson's coefficient tests were performed to assess statistical significance.

Immunohistochemical staining using tissue microarray was further performed and demonstrated that BAG3 intensity was positively with activation of PSCs, as assessed by αSMA staining (Figures 2A,B), and fibrosis, as assessed by Masson staining (Figures 2A,C).

### Reduction of IL6 Production Is Responsible for Suppression of HPanSteC Activation by BAG3 Knockdown

**Figure 3:**
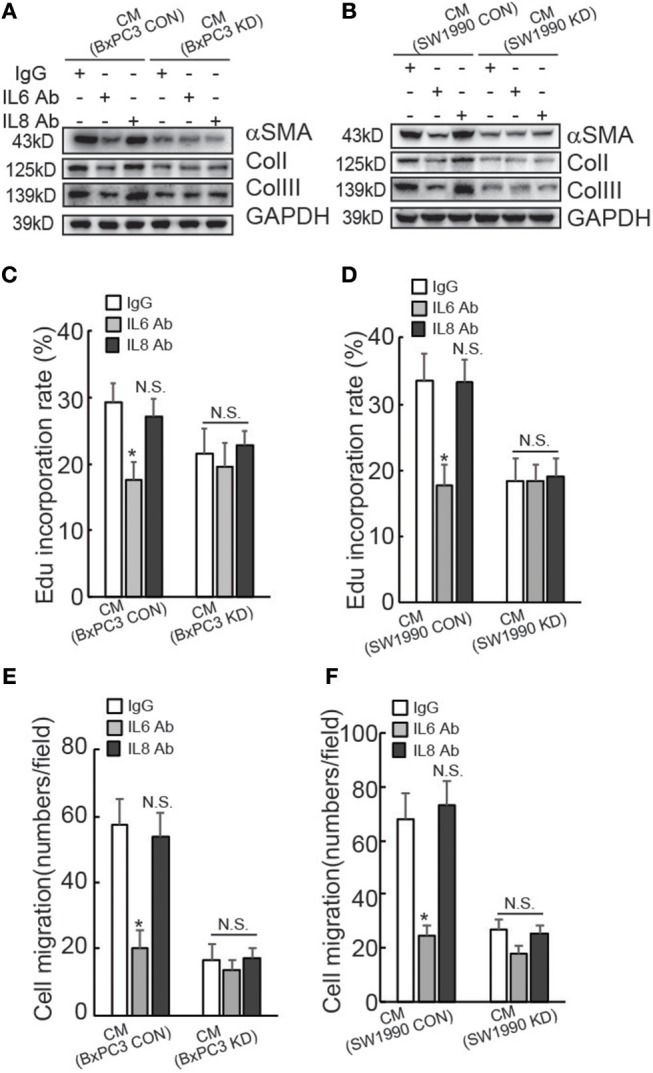
Suppression of IL6 is implicated in suppression of PSCs activation by PDACs with BAG3 knockdown. **(A–F)** CM from PDACs were neutralized with the indicated antibodies, then was used to activate HPanSteC, αSMA, and collagen expression was analyzed using Western blot analysis **(A,B)**, DNA synthesis in the presence of CM from BxPC3 **(C)** or SW1990 **(D)** was analyzed using Edu incorporation, cell migration in the presence of CM from BxPC3 **(E)** or SW1990 **(F)** was analyzed using Transwell. *P < 0.01.

To confirm the involvement of IL6 production in suppression of HPanSteC activation by BAG3 knockdown, neutralizing antibody (Ab) against IL6 was added into CM derived from PDACs. Western blot demonstrated that IL6 Ab significantly decreased αSMA and Collagens expression in HPanSteC cells cultured with CM derived from control PDACs, while had no obvious effect in cells incubated with CM derived from BxPC3 (Figure 3A) or SW1990 (Figure 3B) with BAG3 knockdown. Control IgG or IL8 Ab demonstrated no obvious effect on expression of αSMA and Collagens in HPanSteC cells cultured with CM derived from both control and BAG3 knockdown PDACs (Figures 3A,B). Edu incorporation (Figures 3C,D), and Transwell migration assay (Figures 3E,F) demonstrated that IL6 Ab significantly decreased proliferation (Figures 3C,D) and migration (Figures 3E,F) of HPanSteC cells incubated with CM derived from control PDAC cells, while had little effect on those incubated with CM derived from PDACs with BAG3 knockdown. Control IgG or IL8 Ab had no effect on proliferation (Figures 3C,D) and migration (Figures 3E,F) of HPanSteC cells cultured with CM derived from both control and BAG3 knockdown PDACs.

### Regulation of IL6 mRNA Stability Via its 3′UTR by BAG3 in PDACs

**Figure 4:**
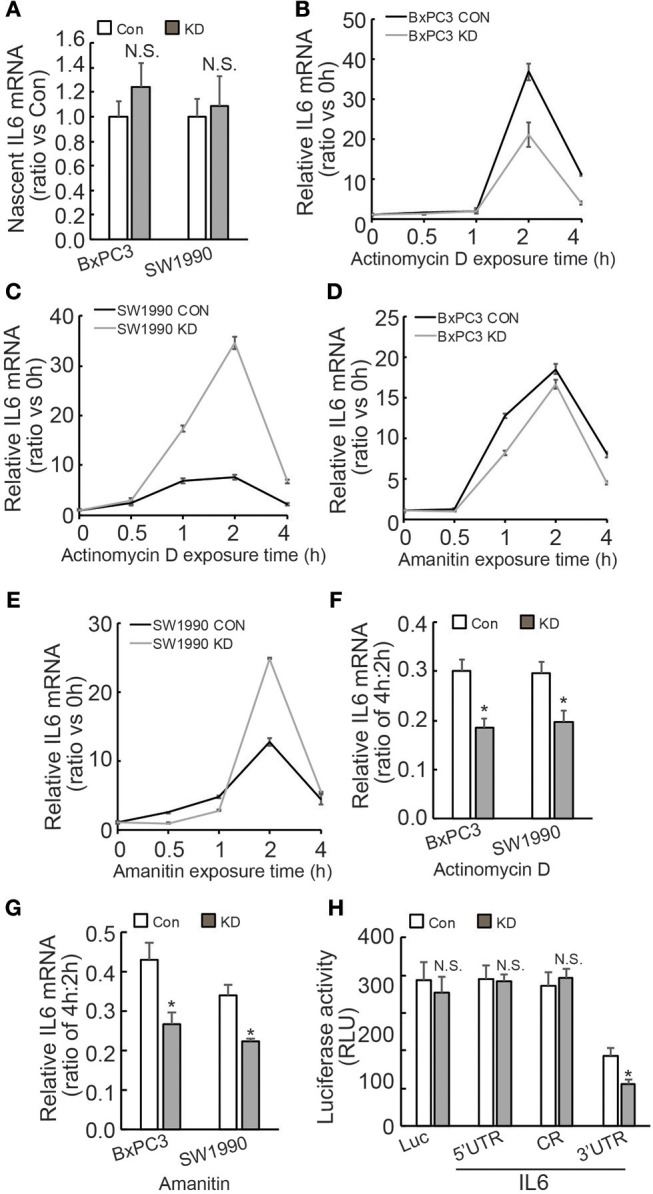
BAG3 knockdown destabilizes IL6 mRNA via its 3′UTR. **(A)** Nascent RNA was labeled and isolated, newly synthesized IL6 mRNA was analyzed using real-time RT-PCR in control or BAG3 knockdown PDAC cells. **(B,C)** Actinomycin D was added for the indicated period to block RNA synthesis, and IL6 mRNA was analyzed using real-time RT-PCR in control or BAG3 knockdown BxPC3 **(B)** or SW1990 **(C)** cells. **(D,E)** Amanitin was added for the indicated period to block RNA synthesis, and IL6 mRNA was analyzed using real-time RT-PCR in control or BAG3 knockdown BxPC3 **(D)** or SW1990 **(E)** cells. **(F,G)** Relative IL6 mRNA expression at 4 h of Actinomycin D **(F)** or Amanitin **(G)** was normalized by that of 2 h. **(H)** Control or BAG3 knockdown BxPC3 cells were transfected with the indicated luciferase reporter vector and a Renilla reporter vector. Luciferase activity was measured 2 days after transfection and Renilla activity was measured to normalize luciferase activity. N.S., not significant; *P < 0.01.

Regulation of IL6 production and involvement of pancreatic fibrosis prompted us to further investigate the mechanisms underlying regulation of IL6 expression by BAG3. Investigation of nascent RNA demonstrated that BAG3 knockdown had no effect on *de novo* IL6 mRNA synthesis (Figure 4A). RNA synthesis inhibitor Actinomycin D was then used to measure the half-life of IL6 mRNA. Unexpectedly, short time exposure of PDACs to Actinomycin D resulted in marked increase in IL6 mRNA levels (Figures 4B,C). Similar phenomena were observed when another RNA synthesis inhibitor Amanitin was used (Figures 4D,E). As the increase reached peak at 2 h of exposure, then decreased abruptly at 4 h (Figures 4B–E), the remained IL6 mRNA at 4 h relative to those at 2 h was calculated and found that remained IL6 mRNA was significantly decreased in PDACs with BAG3 knockdown (Figures 4F,G). To study the cis-acting elements responsible for regulation mediated by BAG3 knockdown, 5′ untranslational region (UTR), CR (coding region), as well as 3′UTR of *IL6* gene was inserted just after the stop codon of luciferase (Luc) gene. The luciferase activity was significantly decreased when the 3′UTR of IL6 was inserted into the reporter construct (Figure 4H). In addition, BAG3 knockdown further significantly decreased the luciferase activity of reporter construct containing IL6 3′UTR (Figure 4H). BAG3 knockdown did not alter the luciferase activity of control reporter construct, or reporter constructs with insertion of 5′UTR or CR (Figure 4H).

### BAG3 Knockdown Destabilizes IL6 mRNA in PDACs Through Ago2 Dependent Mechanism

**Figure 5:**
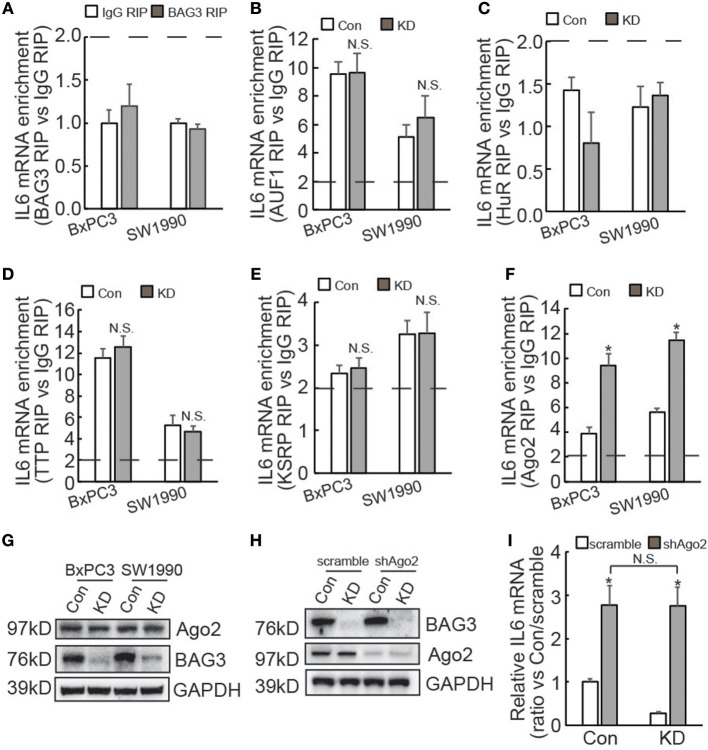
BAG3 knockdown destabilizes IL6 mRNA in a miRNA-depdendent manner. **(A–F)** RIP was performed using BAG3 **(A)**, AUF1 **(B)**, HuR **(C)**, TTP **(D)**, KSRP **(E)**, or Ago2 **(F)** antibody with lysates from control or BAG3 knockdown PDAC cells. IL6 mRNA enrichment was analyzed using real-time RT-PCR. **(G)** Ago2 expression was analyzed using Western blot analysis. **(H)** Ago2 was knocked down using lentivirus containing shRNA against Ago2 (shAgo2), knockdown efficiency was confirmed using Western blot. **(I)** and IL6 mRNA expression was analyzed using real-time RT-PCR. N.S., not significant; *P < 0.01.

IL6 3′UTR contains multiple AU-rich elements (AREs), which are directly targeted by several ARE binding proteins, including AUF1 (35), HuR (36), TTP (37), and KSRP (38). In addition, we have recently reported that BAG3 *per se* is recruited to some mRNAs and regulates their stability (25, 39, 40). RIP demonstrated that BAG3 did not interact with the IL6 transcript (Figure 5A). In addition, BAG3 knockdown did not affect recruitment of AUF1 (Figure 5B), HuR (Figure 5C), TTP (Figure 5D), or KSRP (Figure 5E) to the IL6 transcript in PDAC cells. As non-coding RNAs also play critical roles in the post-transcriptional regulation of gene expression via their recruitment to the 3′UTR of target mRNAs (41), RIP using antibody against Ago2 was then performed. More than 2-fold enrichment of IL6 mRNA by Ago2 was observed in control BxPC3 and SW1990 cells (Figure 5F), indicating that IL6 mRNA might be a target for miRNA-mediated gene silencing (miRISC) in PDACs. Importantly, BAG3 knockdown significantly promoted recruitment of Ago2 to IL6 mRNA in both BxPC3 and SW1990 cells (Figure 5F). Western blot demonstrated that Ago2 expression was unaltered by BAG3 knockdown in PDACs (Figure 5G). To investigate the potential involvement of miRISC on destabilization of IL6 mRNA by BAG3 knockdown, Ago2 expression was knocked down using lentivirus containing shRNAs against Ago2 (shAgo2) in BxPC3 cells (Figure 5H). Ago2 knockdown significantly increased IL6 mRNA expression in both control and BAG3 knockdown cells (Figure 5I). It should be noted that BAG3 knockdown cells expressed similar extent of IL6 mRNA to control cells, when Ago2 was downregulated (Figure 5I).

### Ago2 Phosphorylation at Ser387 Is Required for Its Loading to IL6 mRNA and BAG3 Knockdown Promotes Ago2 Recruitment to IL6 mRNA via Increasing Its Ser387 Phosphorylation

**Figure 6:**
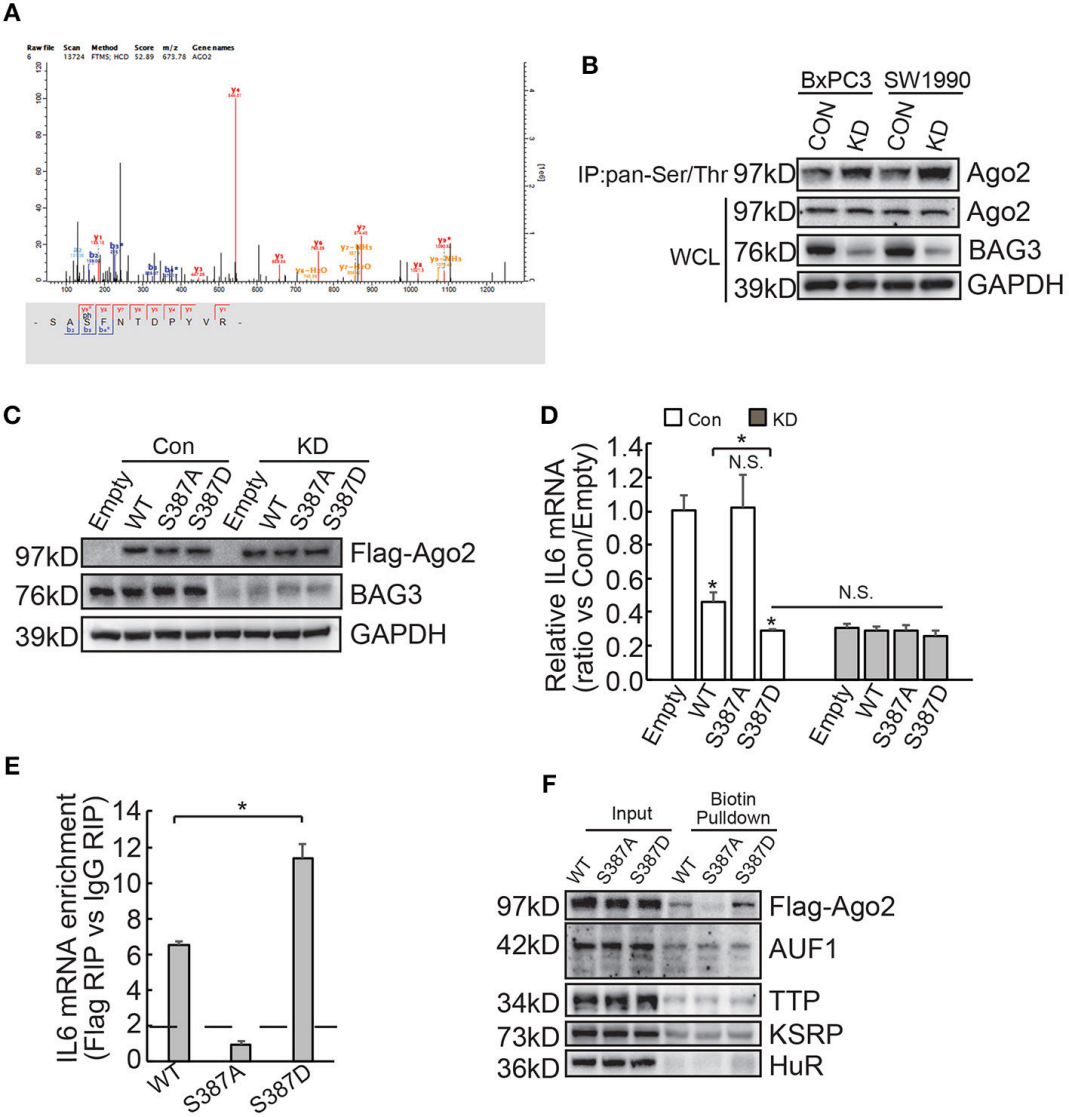
BAG3 knockdown increases Ago2 phosphorylation at Ser387 and facilitates recruitment of Ago2 to IL6 mRNA. **(A)** Matrix assisted laser desorption ionization time-of-flight peptide mass spectrum showed that phosphorylation of Ago2 at Ser387 was increased in BAG3 knockdown BxPC3 cells. **(B)** Immunoprecipitation was performed using pan-Ser/Thr antibody, Ago2 phosphorylation was then analyzed using Western blot analysis. **(C–E)** BxPC3 cells were transfected with wild type (WT), mutation at Ser387 to alanine (S387A) or to aspartic acid (S387D) Ago2, Ago2 expression was confirmed by Western blot **(C)**, IL6 mRNA was analyzed using real-time RT-PCR **(D)**, enrichment of IL6 mRNA by Ago2 was performed using RIP with Flag antibody followed by real-time RT-PCR **(E)**. **(F)** Biotinylated 3′UTR RNA segment of IL6 mRNA was used to pulldown lysates from BxPC3 cells transfected with WT, S387A or S387D Ago2 construct, and the pulldown materials were analyzed by western blotting analysis using the indicated antibodies. N.S., not significant; *P < 0.01.

Differential expression of miRNAs by BAG3 knockdown was then explored and Genechip data demonstrated that none of differentially expressed miRNAs appear to target IL6 mRNA (Supplementary Data 2). We explored phosphorylation proteomics regulated by BAG3 using quantitative proteomics technology and found that BAG3 knockdown increased phosphorylation of Ago2 at Ser387 site in BxPC3 cells (Figure 6A). Immunoprecipitation using pan-Ser/Thr phosphorylation antibody followed by Western blot confirmed that BAG3 knockdown increased Ago2 phosphorylation in PDACs (Figure 6B). BxPC3 cells were then transfected with constructs containing wild-type (WT), non-phosphorylatable mutant (S387A), or phosphorylation mimetic mutant (S387D) Ago2 (Figure 6C). Real-time PCR demonstrated that WT and S387D Ago2 significantly decreased, while S387A Ago2 had no effect on IL6 mRNA expression in control BxPC3 cells (Figure 6D). None of Ago2 had effect on IL6 mRNA expression in BxPC3 cells with BAG3 knockdown (Figure 6D). It should be noted that S387D Ago2 demonstrated more potent suppressive effect when compared with WT Ago2 (Figure 6D). In addition, BAG3 knockdown cells expressed comparable IL6 mRNA with their control partner when S387D Ago2 were ectopically expressed (Figure 6D). RIP demonstrated that WT and S387D Ago2 effectively immunoprecipitated IL6 mRNA, while IL6 mRNA was not enriched by S387A Ago2 (Figure 6E). In addition, the extent of enrichment by S387D Ago2 was greater than that by WT Ago2 (Figure 6E). Biotin pulldown also demonstrated that both WT and S387D Ago2 was pulled down by biotinylated 3′UTR fragment of IL6 mRNA, while S387A Ago2 was undetectable in pulldown materials (Figure 6F). Binding of AUF1, TTP, KSRP, or HuR with 3′UTR fragment of IL6 mRNA was unaltered by phosphorylation status of Ago2 (Figure 6F).

## Discussion

The presence of dense desmoplastic stroma is a hallmark of PDAC, forming a unique microenvironment that provides survival and proliferative signals to promote tumor initiation, progression and therapy resistance. Thus, modulation of the stroma response is considered a promising opportunity for the PDAC therapy (42, 43). PSCs compose a critical cellular component of pancreatic cancer stroma, which reside in a quiescent state in the normal pancreas, but transform to an activated state in cancer microenvironment. Activated PSCs proliferate at a high rate and play a key role in pancreatic fibrosis. Many studies have reported a high level of BAG3 expression in cancer cells, and this has been associated with poor prognosis in patients with PDACs (34). However, the pathophysiological mechanisms are not completely clarified yet. In the current study, we demonstrated that BAG3 expression positively correlated with fibrosis in PDAC tissues. In addition, downregulation of BAG3 in PDACs significantly decreased their ability to activate PSCs.

Although the exact factors or means by which cancer cells trigger quiescent PSCs to transit into an activated phenotype are not entirely understood yet, it is well established that paracrine, as well as autocrine mediators, play a critical role in the maintenance of PSC activation in pancreatic cancer (44). One of notable examples of such mediators is IL6, which may function in an autocrine and paracrine pattern (30, 45). In the current study, we demonstrated that both BxPC3 and SW1990 cells secreted a large amount of IL6, while knockdown of BAG3 significantly decreased IL6 release by BxPC3 and SW1990 cells.

The current study demonstrated that BAG3 knockdown decreased total IL6 mRNA levels, while had no obvious effect on newly synthesized IL6 mRNA levels, indicating that BAG3 knockdown might destabilize IL6 mRNA at the post-transcriptional level. Unexpectedly, we observed a dramatic increase in the level of IL6 mRNA within 2 h after addition of transcriptional inhibitors. Super-induction by transcriptional inhibitors was also observed in P-glycoprotein (P-gp) mRNA (46). The current study demonstrated that RNA synthesis inhibitors Actinomycin D and α-Amanitine did not decrease, but even increased IL6 mRNA during the first 2 h exposure. Consistent with our findings, it has been reported super-induction of IL6 in peritoneal mesenchymal cells (47) and macrophages (48). One possible explanation for such super-induction is that the transcriptional inhibitors directly or indirectly suppress degradation of normally extremely unstable mRNAs. The current study found that super-induction of IL6 mRNA by transcriptional inhibitors was enhanced by BAG3 knockdown in SW1990 cells, while such a phenomenon was not observed in BxPC3 cells. These data indicated that BAG3 might be implicated in super-induction of IL6 mRNA in a cell context-dependent manner. Irrespective of BAG3 expression levels, super-induction of IL6 mRNA reached the maximum about 2 h after addition of transcriptional inhibitors. BAG3 knockdown promoted degradation of IL6 mRNA after maximal accumulation in PDACs, confirming that BAG3 knockdown decreased stability of IL6 mRNA.

Very recently, our lab found that BAG3 regulated stability of some mRNAs via interaction (25, 39, 40). However, the current study demonstrated that BAG3 *per se* was not recruited to IL6 mRNA using both RIP and biotin pulldown experiments. The current study identified that 3′UTR of IL6 mRNA was responsible for regulation by BAG3. Several ARE binding proteins are reported to directly target IL6 mRNA via AREs located on its 3′UTR (35, 36, 49). The current study demonstrated that recruitment of ARE binding proteins including AUF1, HuR, TTP, and KSRP was unaltered by BAG3 knockdown. On the contrary, we found that recruitment of Ago2 to IL6 mRNA was significantly increased in PDACs with BAG3 downregulation. In addition, the current study demonstrated that Ago2 was responsible for destabilization of IL6 mRNA by BAG3 downmodulation. None of differentially expressed miRNAs screened between control and BAG3 knockdown BxPC3 cells seemed to target IL6 mRNA, indicating that alternative mechanism(s) underlying destabilization of IL6 mRNA by BAG3 knockdown.

The current study demonstrated that BAG3 knockdown increased Ago2 phosphorylation at Ser387 site. In addition, we found that WT and phosphorylation mimic S387D Ago2, but not non-phosphorylatable S387A Ago2 significantly enriched and destabilized IL6 mRNA in PDACs, suggesting that phosphorylation of Ago2 at Ser387 might play a critical role in its recruitment to IL6 mRNA. It has been reported that Ago2 phosphorylation at Ser387 plays a critical role in mediating its distribution to processing bodies (PBs) under normal conditions (50), while reduces its secretion into exosomes and specifically controls let-7a, miR-100, and miR-300a levels in exosomes (51). In addition, Akt-mediated phosphorylation of Ago2 at S387 diverts its activity from mRNA degradation to translational repression of miRNA targets (52). These reports indicated that phosphorylation of Ago2 at Ser387 site might play a critical role in regulating its localization, as well as loading to both miRNA and mRNA targets.

**Figure 7:**
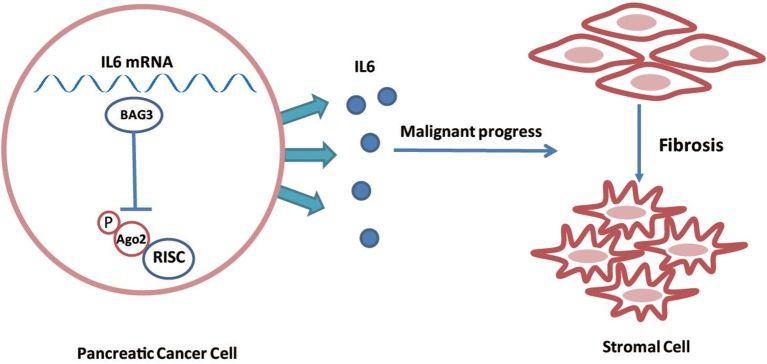
Schematic representation of that BAG3 expression levels in PDACs regulate their ability to drive PSCs activation via regulating stability of IL6 mRNA.

In conclusion, the current study demonstrated that BAG3 expression levels in PDACs regulate their ability to drive PSCs activation via regulating stability of IL6 mRNA (Figure 7). In addition, the current study demonstrated that phosphorylation of Ago2 at Ser 387 plays an essential role on its loading to IL6 mRNA. The involvement of BAG3 in PSCs activation highlights the potential BAG3 as a therapeutic target for preventing PDAC progression, as a concept of modulating the activation of PSCs in the context of fibrosis.

## Data Availability

All datasets generated for this study are included in the manuscript and/or the Supplementary Files.

## Author Contributions

H-QW and CL designed experiments. CL, M-XA, J-YJ, H-BY, SL, JY, and X-YL carried out experiments. CL and H-QW analyzed experimental results. M-XA and J-YJ developed analysis tools. CL wrote the manuscript.

### Conflict of Interest Statement

The authors declare that the research was conducted in the absence of any commercial or financial relationships that could be construed as a potential conflict of interest.
